# No-Reference Image Quality Assessment Based on the Fusion of Statistical and Perceptual Features

**DOI:** 10.3390/jimaging6080075

**Published:** 2020-07-30

**Authors:** Domonkos Varga

**Affiliations:** Department of Networked Systems and Services, Budapest University of Technology and Economics, H-1111 Budapest, Hungary; varga.domonkos7@upcmail.hu

**Keywords:** no-reference image quality assessment, fractal dimension, Benford’s law

## Abstract

The goal of no-reference image quality assessment (NR-IQA) is to predict the quality of an image as perceived by human observers without using any pristine, reference images. In this study, an NR-IQA algorithm is proposed which is driven by a novel feature vector containing statistical and perceptual features. Different from other methods, normalized local fractal dimension distribution and normalized first digit distributions in the wavelet and spatial domains are incorporated into the statistical features. Moreover, powerful perceptual features, such as colorfulness, dark channel feature, entropy, and mean of phase congruency image, are also incorporated to the proposed model. Experimental results on five large publicly available databases (KADID-10k, ESPL-LIVE HDR, CSIQ, TID2013, and TID2008) show that the proposed method is able to outperform other state-of-the-art methods.

## 1. Introduction

Visual signals (digital images or videos) undergo a wide variety of distortions during acquisition, compression, transmission, and storage. Thus, image quality assessment (IQA) is crucial to predict the quality of digital images in many applications, such as compression, communication, printing, display, analysis, registration, restoration, and enhancement [1,2,3,4]. Generally, it can be used in benchmarking any image processing algorithms. Furthermore, it is indispensable in evaluating any new hardware or software component related to imaging. For instance, a number of biometric algorithms rely on images, such as palmprint, fingerprint, face image, or handwriting recognition. However, the acquisition process of images is often not perfect in practice. Because of this, information about the quality degradation of images is required [5]. Similarly, the performance of object detection (e.g., pedestrian, car, traffic sign, etc.) algorithms heavily depends on the image quality [6,7]. As a consequence, monitoring image/video quality is also crucial in vision-based advanced driver-assistance systems [8].

Since human observers are the end users of visual content, the quality of visual signals is ideally evaluated by subjective user studies in a laboratory environment involving specialists. During these user studies, subjective quality scores are collected from each participant. Subsequently, the quality of a visual signal is given by mean opinion score (MOS), which is defined as the arithmetic mean of the individual ratings. In most cases, an absolute category rating (ACR) scale is applied where the range usually goes from 1.0 (very poor quality) to 5.0 (excellent quality). Several international standards such as ITU BT.500-13 [9] or ITU P910 [10] have been proposed for performing subjective visual quality assessment. As already mentioned, the main goal of subjective visual quality assessment is to assign a score of the users’ perceived quality to each visual signal in a given set of signals (images or videos). However, the resulted assessment might vary significantly because of many factors such as lighting conditions and the choice of the subjects. That is why ITU-R BT.500-13 [9] gives detailed recommendations about viewing conditions, monitor resolution, selection of test materials, observers, test session, grading scales, analysis and interpretation of the results.

Subjective visual quality assessment has some drawbacks which limit their applications. Namely, they are time-consuming and expensive because subjective results are obtained through experiments with many observers. As a consequence, they cannot be part of real-time applications such as image transmission systems. Therefore, the development of objective visual quality assessment methods that are able to predict the perceptual quality of visual signals is of high importance. The classification of objective visual quality assessment algorithms is based on the availability of the original (reference) signal. If a reference signal is not available, a visual quality assessment algorithm is regarded as a no-reference (NR) one. NR algorithms can be classified into two further groups, where the so-called distortion-specific NR algorithms assume that a specific distortion is present in the visual signal, whereas general purpose (or non-distortion specific) algorithms operate on various distortion types. Reduced-reference (RR) methods retain only part of the information from the reference signal, whereas full-reference (FR) algorithms have full access to the complete reference medium to predict the quality scores. Similar to NR methods, FR algorithms can be also classified into distortion-specific and general purpose ones. The research of objective visual quality assessment demands databases that contain images or videos with the corresponding MOS values. To this end, a number of image or video quality databases have been made publicly available. According to the database structure, these databases can be categorized into three groups. The first one contains a smaller set of pristine, reference visual signals and artificially distorted images derived from the pristine visual signals considering different artificial distortions at different intensity levels. The second group contains only digital images or videos with authentic distortions collected from photographers, so pristine images or videos cannot be found in such databases. As a consequence, the development of FR methods is connected to the first group of databases. In contrast, NR-IQA algorithms can be trained and tested on both types of databases.

The rest of this section is organized as follows. In Section 1.1, the related and previous work in NR-IQA are reviewed. The main contributions of this study are declared in Section 1.2. The structure of this paper is described in Section 1.3.

### 1.1. Related Work

Previous and related work are introduced in this section, including a brief review of distortion-specific, opinion aware, and opinion unaware methods. The so-called distortion-specific methods presume one or several types of distortions in the image, such as blur [11], blockiness [12], ringing [13], or JPEG2000 noise [14]. The disadvantage of this line of work is quite obvious. Namely, the number of possible image distortion and noise types is large, while they are able to consider only several noise types. In contrast, general opinion aware methods are trained on features extracted from distorted images to predict perceptual quality over various types of image distortions. For example, the blind image quality indices (BIQI) [15] method contains two stages. First, the distortion present in the image is determined with the help of a trained support vector machine (SVM) [16] given a set of image noise types. Second, the perceptual quality is evaluated with respect to the distortion. Numerous opinion aware methods are based on the so-called natural scene statistics (NSS) which is a subfield of perception and deals with the statistical regularities of natural scenes. More specifically, it is assumed that natural images have certain statistical regularities which are biased by visual distortions. That is why, NSS have become successful in perceptual image and video quality prediction. For instance, Saad et al. [17] devised a statistical model in the discrete cosine transform (DCT) domain. Specifically, it utilizes a Bayesian approach to predict perceptual quality based on a set of DCT coefficients related features. In contrast, Lu et al. [18] assumed that image distortions can be characterized in the wavelet domain. On the other hand, Lu et al. [19] developed an NSS model in the contourlet domain. Namely, the statistics of contourlet coefficients was used to estimate image quality. In blind/referenceless image spatial quality evaluator [20] (BRISQUE), scene statistics of locally normalized luminance coefficients are applied to train a support vector regressor [21] (SVR) for perceptual quality prediction. In the spatial domain, image gradient magnitude have been used by many researchers to predict image quality [22,23,24]. Specifically, Xue et al. [23] utilizes the joint local contrast features from the gradient magnitude map and the Laplacian of Gaussian response. In feature maps based referenceless image quality evaluation engine (FRIQUEE) [25], a large set of features is defined using perceptually relevant color and transform domain spaces. Liu et al. [26] extracted features from the distorted images’ curvelet domain and trained an SVM for perceptual quality prediction. In contrast, Li et al. [27] extracted features from the distorted images with the help of shearlet transform. Subsequently, stacked autoencoders were applied to make these features more discriminative. Finally, a softmax classifier was used for quality prediction. Ou et al. [28] were the first who applied Benford’s law in image quality assessment. Namely, they pointed out features based on Benford’s law are very sensitive to white noise, Gaussian blur, and fast fading. Freitas et al. [29] combined the statistics of different color and texture descriptors and mapped onto quality scores using a gradient boosting machine. In an other study, Freitas et al. [30] compared the performance of different local binary pattern texture descriptors for NR-IQA.

Opinion unaware methods require neither training images of distortions nor perceptual quality scores. For example, Mittal et al. [31] extracted BRISQUE [20] features from image patches and perceptual quality was defined as the distance between the NSS-based features extracted from the test image to the features obtained from the target IQA database. Moreover, these features were approximated by multivariate Gaussian distributions. This method was further developed by Zhang et al. [32] who incorporated more quality aware features and a local quality measurement procedure into the previous model. In contrast, Xue et al. [33] proposed a quality-aware clustering strategy to determine a set of cluster centroids. Next, these centroids were utilized as a codebook to estimate perceptual image quality.

### 1.2. Contributions

The main contributions of this study are as follows. An NR-IQA method is introduced which is driven by a novel feature vector. Furthermore, it contains new elements which cannot be found in previous methods. Namely, this is the first work that applies local fractal dimension distribution of an image for NR-IQA. Although Ou et al. [28] derived first quality aware features with the help of Benford’s law, the proposed method directly measures the first digit distribution in the wavelet and spatial domain to define features. Moreover, the above mentioned novel statistical features are enriched with powerful perceptual features, such as colorfulness, global contrast factor, dark channel feature, entropy, and mean of phase congruency image. Experimental results on five large publicly available quality databases show that the proposed method is able to significantly outperform other state-of-the-art methods. This paper is accompanied by the source code of the proposed method (https://github.com/Skythianos/SPF-IQA).

### 1.3. Structure

This study is organized as follows. After this introduction, Section 2 describes the proposed IQA method. In Section 3, the evaluation metrics, the experimental setup, a parameter study, and a comparison to other state-of-the-art methods are given. Finally, a conclusion is drawn in Section 4.

## 2. Methodology

**Figure 1:**
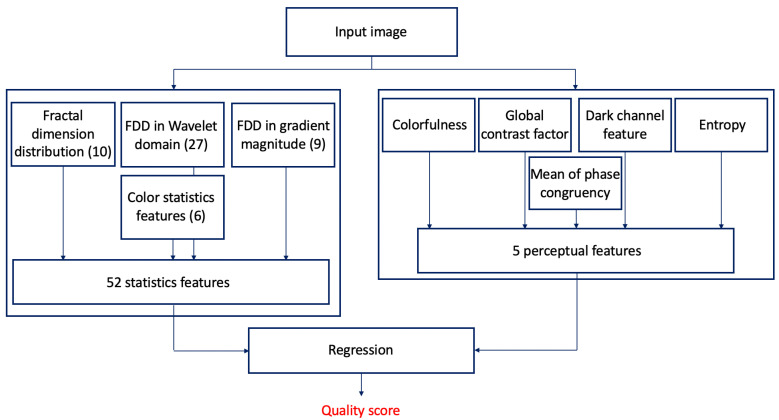
Architecture of the proposed no-reference image quality assessment method.

The general overview of the proposed method is depicted in Figure 1. Statistics and perceptual features are extracted from the input image which are mapped onto perceptual quality scores with the help of a regression technique. Specifically, the statistics features are used to capture the differences between the statistical patterns of pristine, natural images and those of distorted images. To this end, the fractal dimension distribution, the first digit distribution in the wavelet and gradient magnitude domain, and color statistics features are extracted. Since some perceptual features consistent with human quality judgements, the following perceptual features are incorporated into the proposed model: colorfulness, global contrast factor, dark channel feature, entropy, and mean of phase congruency image.

The rest of this section is organized as follows. The proposed statistical features are introduced in Section 2.1, while the used perceptual features are described in Section 2.2.

### 2.1. Statistical Features

**Figure 2:**
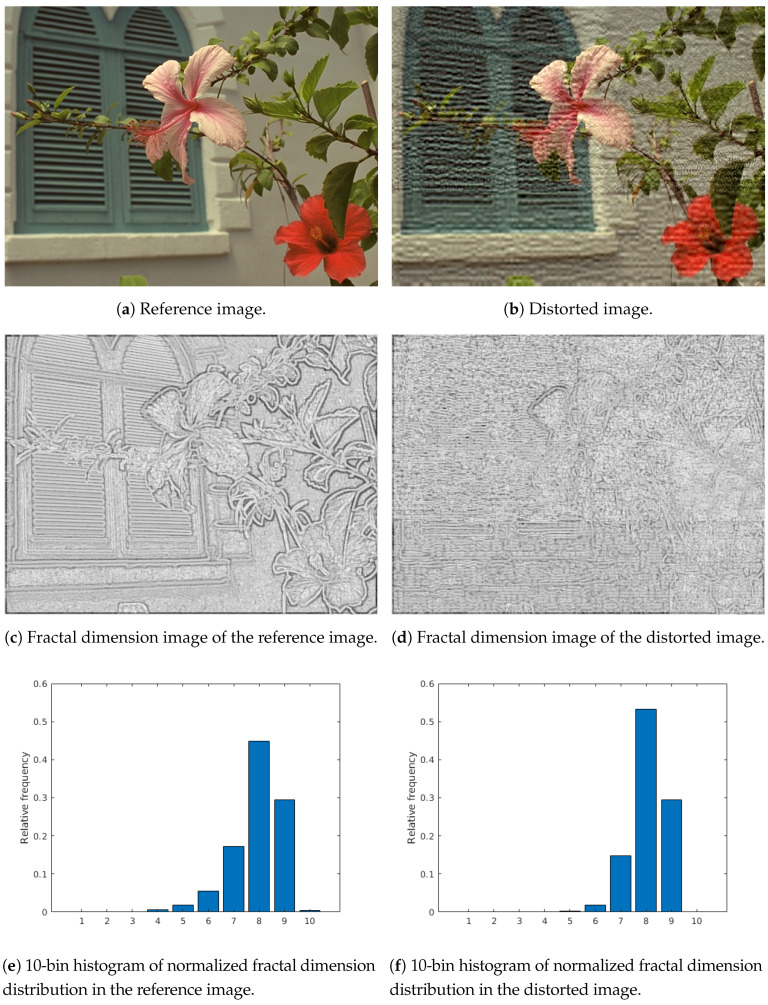
Illustration to fractal dimension distribution. Fractal dimension images are produced by considering each pixel in the original image as a center of a 7 × 7 patch and the fractal dimension is calculated from this patch. Furthermore, black pixels correspond to −2 fractal dimension, while white ones correspond to +3.

**Figure 3:**
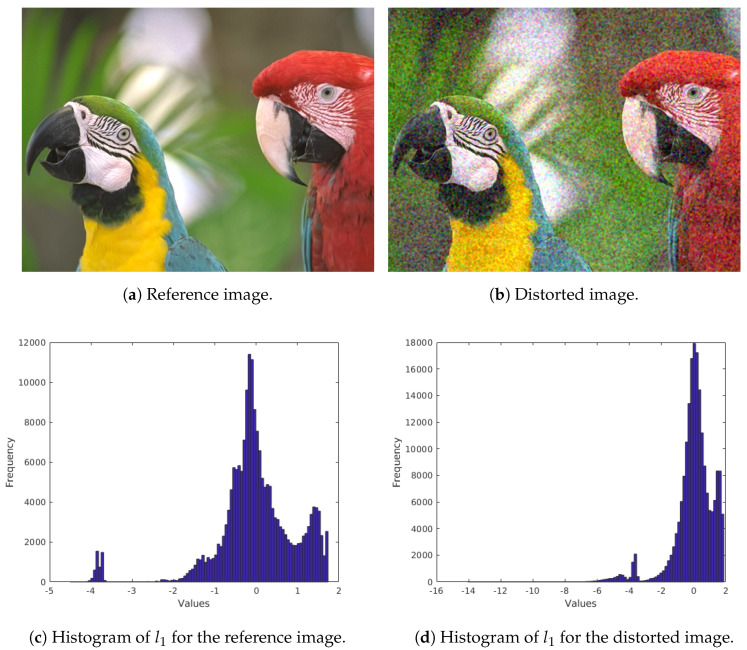
Illustration of *l*_1_ values distribution in a reference and a distorted image from TID2013 [39] database.

1. Local fractal dimension distribution: Fractal analysis was first proposed by Mandelbrot [34] and it deals with the study of irregular and self-similar objects. By definition, fractal dimenstion characterizes patterns or sets “by quantifying their complexity as a ratio of the change in detail to the change in scale” [34]. The fractal dimension image is produced by considering each pixel in the original image as a center of a 7-by-7 rectangular neighborhood and the fractal dimension is calculated from this neighborhood. To determine the fractal dimension of a grayscale image patch, the box counting technique developed by Al-Kadi and Watson [35] was applied. From the fractal dimension image, a 10-bin normalized histogram was calculated considering the values between −2 and 3. Figure 2 illustrates the local fractal dimension images of a reference and a distorted image. It can be observed that the fractal dimension of an image patch is extremely sensitive to image distortions.
2. First digit distribution in wavelet domain: Benford’s law, also called the first digit law, states leading digit d(d∈1,…,9) in many real-world datasets occurs with probability
  (1) $$P\left(d\right)=\log_{10}\left(d+1\right)-\log_{10}\left(d\right)=\log_{10}\left(\frac{d+1}{d}\right)=\log_{10}\left(1+\frac{1}{d}\right).$$
  More specifically, Benford’s law [36] works on a distribution of numbers if that distribution spans quite a few order of magnitudes. As pointed out in [37], the first digit distribution in the transform domain of a pristine natural image harmonizes better with the Benford’s law than those of a distorted image. In this study, the normalized first digit distribution is utilized in the wavelet domain and in the image gradient domain to extract feature vectors. Specifically, an Fejér-Korovkin wavelet [38] was used to transform the image into wavelet domain. Next, the normalized first digit distribution was measured in horizontal detail coefficients, vertical detail coefficients, and diagonal detail coefficients. Finally, a 27-dimensional feature vector was obtained in the wavelet domain by concatenating the normalized first digit distributions in the horizontal, vertical, and diagonal detail coefficients, respectively.
3. First digit distribution in gradient magnitude: The gradient of the image was determined with the help of 3-by-3 Sobel operator. The normalized first digit distribution of the gradient magnitude image was measured and a 9-dimensional feature vector was compiled.
  Table 1 illustrates the average and median Euclidean distances between first digit distributions of TID2013 [39] images in the wavelet and gradient magnitude domain and Benford’s law prediction. It can be observed that the first digit distributions in the wavelet domain is almost the same as Benford’s law prediction. Moreover, it can be clearly seen that the distorted images distance from Benford’s law is significantly larger than those of the reference images. One can further observe that the most distorted images’ first digit distribution Euclidean distance from the Benford’s law is the largest, since the standard deviation of the distance values for the distorted images is three times larger than those of the reference images. In the gradient magnitude domain, the above mentioned observations are less significant. Furthermore, the standard deviations are almost the same.
4. Color statistics features: To extract the statistical properties of the color, the model of Ruderman et al. [40] was applied. Specifically, an RGB image was first transformed into a mean-subtracted logarithmic signal:
  (2) $$R_{1}\left(i,j\right)=\log\left(R\left(i,j\right)\right)-\mu_{R},$$
  (3) $$G_{1}\left(i,j\right)=\log\left(G\left(i,j\right)\right)-\mu_{G},$$
  (4) $$B_{1}\left(i,j\right)=\log\left(B\left(i,j\right)\right)-\mu_{B},$$
  where $\mu_{R}$, $\mu_{G}$, and $\mu_{B}$ are the mean values of the logarithms of the *R*, *G*, and *B* image channels, respectively. From these signals, the following $l_{1}$, $l_{2}$, and $l_{3}$ signals are derived:
  (5) $$l_{1}\left(x,y\right)=\frac{\left(R_{1}+G_{1}+B_{1}\right)}{\sqrt{3}},$$
  (6) $$l_{2}\left(x,y\right)=\frac{\left(R_{1}+G_{1}-2B_{1}\right)}{\sqrt{6}},$$
  (7) $$l_{3}\left(x,y\right)=\frac{\left(R_{1}-G_{1}\right)}{\sqrt{2}}.$$
  As pointed out by Ruderman et al. [40], the distributions of the coefficients in $l_{1}$, $l_{2}$, and $l_{3}$ approximately fit to Gaussian distributions for natural images (see Figure 3 for an example). As a consequence, a Gaussian distribution was fit to the coefficients of $l_{1}$, $l_{2}$, and $l_{3}$. Moreover, the mean and the variance were taken as quality-aware features. As a result, the color statistics feature vector contains six elements (mean and variance for $l_{1}$, $l_{2}$, and $l_{3}$). Figure 3 illustrates the distribution of $l_{1}$ values in a reference image and in its distorted counterpart from TID2013 [39] database.

### 2.2. Perceptual Features

**Figure 4:**
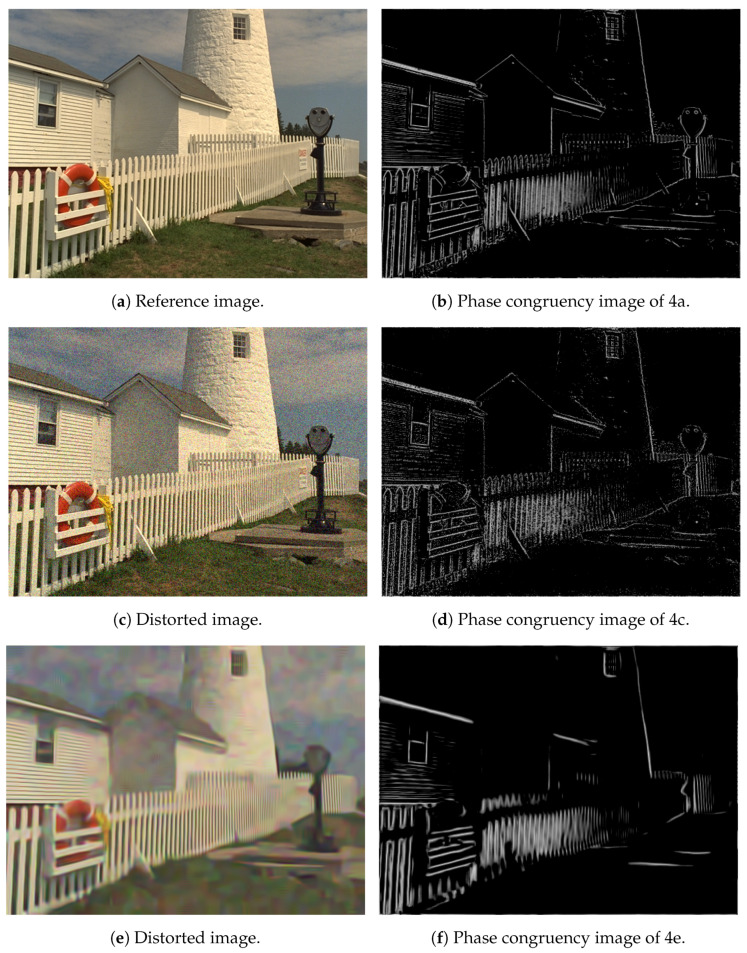
Illustration of phase congruency.

1. Colorfulness: As pointed out in [41], humans prefer slightly more colorful images and colorfulness influence perceptual quality judgements. It was calculated using the following formula [42]:
  (8) $$CF=\sqrt{\sigma_{rg}^{2}+\sigma_{yb}^{2}}+\frac{3}{10}\sqrt{10\mu_{rg}^{2}+\mu_{yb}^{2}},$$
  where σ and μ stand for the standard deviation and mean of the matrices given in the subscripts. Furthermore, rg=R−G and $yb=\frac{1}{2}\left(R+G\right)-B$, where *R*, *G*, and *B* denote the red, green, and blue channels of the input image, respectively.
2. Global contrast factor: Humans’ ability to recognize or distinguish objects in the image strongly depends on the contrast. As a consequence, contrast may influence the perceptual quality and is incorporated into the proposed model. In this study, Matkovic et al.’s [43] model was applied which is limited to grayscale contrast. Global contrast factor is computed as follows:
  (9) $$GCF=\sum_{i=1}^{9}w_{i}C_{i},$$
  where $w_{i}$ is defined as $w_{i}=\left(-0.406385\cdot \frac{i}{9}+0.334573\right)\cdot \frac{i}{9}+0.0877526$, i∈{1,2,…,9}. Furthermore, $C_{i}$ is
  (10) $$C_{i}=\frac{1}{w\cdot h}\sum_{i=1}^{w\cdot h}l_{C_{i}},$$
  and
  (11) $$l_{C_{i}}=\frac{|L_{i}-L_{i-1}\left|+\right|L_{i}-L_{i+1}\left|+\right|L_{i}-L_{i-w}\left|+\right|L_{i}+L_{i+w}|}{4},$$
  where *L* stands for the intensity pixel value of the image after applying gamma correction (γ=2.2). Assuming that the image’s width is *w* and its height is *w*, and the image is reshaped into a row-wise one dimensional array.
3. Dark channel feature: He et al. [44] called dark pixels those pixels whose intensities in at least one color channel are very low. Specifically, a dark channel was defined as:
  (12) $$I^{dark}\left(x\right)=\min_{y\in \Omega (x)}\left(\min_{c\in R,G,B}I^{c}\left(y\right)\right),$$
  where $I^{c}$ denotes the intensity value for a color channel c∈{R,G,B} and Ω(x) stands for the image patch centered around pixel *x*. In this study, the dark channel feature of an image is defined as:
  (13) $$DCF=\frac{1}{\left||S|\right|}\sum_{i\in S}\frac{I^{dark}\left(i\right)}{\sum_{c\in R,G,B}I^{c}\left(i\right)},$$
  where $I^{c}$ denotes the intensity value for a color channel c∈R,G,B and Ω(x) stands for an image patch centered around pixel *x*. Moreover, *S* denotes the area of the input image.
4. Entropy: It has many different interpretations, such as “measure of order” or “measure of randomness”. In other words, it describes how much information is provided by the image. Therefore, it can be applied to characterize the texture of an image. Furthermore, image entropy changes with the type and level of image distortions. Entropy of a grayscale image I is defined as:
  (14) $$E_{I}=-\sum_{n}p\left(n\right)\cdot \log_{2}p\left(n\right),$$
  where p(n) is the empirical distribution of the grayscale values.
5. Mean of phase congruency image: The main idea behind phase congruency is that perceptually significant image features can be observed at those spatial coordinates where the Fourier series components are maximally in phase [45]. The formal definition of phase congruency (PC) is the following [46],
  (15) $$PC_{1}\left(x\right)=\frac{\left|E(x)\right|}{\sum_{n}A_{n}\left(x\right)},$$
  where E(x) is the energy of signal *x* and $A_{n}\left(x\right)$ stands for the *n*th Fourier amplitude. Equation (15) was developed further by Kovesi [45] by incorporating noise compensation,
  (16) $$PC_{2}\left(x\right)=\frac{\sum_{n}W\left(x\right)⎣A_{n}\left(x\right)\Delta \varphi_{n}\left(x\right)-T⎦}{\sum_{n}A_{n}\left(x\right)+\varepsilon },$$
  where W(x) weights for frequency spread, and ⎣·⎦ denotes the floor function, *T* is an estimation for the noise level, and ε is a small constant for avoiding the division by 0. Furthermore,
  (17) $$\Delta \varphi_{n}\left(x\right)=cos\left(\varphi_{n}\left(x\right)-\bar{\varphi }\left(x\right)\right)-\left|sin\left(\varphi_{n}\left(x\right)-\bar{\varphi }\left(x\right)\right)\right|,$$
  where $\varphi_{n}\left(x\right)$ is the phase of the *n*th Fourier component at *x* and $\bar{\varphi }\left(x\right)$ is the average phase at *x*. Phase congruency was used to detect boundary, texture direction, and image segmentation [47]. In Figure 4, it is illustrated that perceptual quality degradations severely modify the phase congruency image. That is why, the mean of the phase congruency image was used as a perceptual metric in this study.

## 3. Experimental Results

In this section, experimental results and analysis are presented. Specifically, the evaluation protocol is given in Section 3.1. The experimental setup is described in Section 3.2. A parameter study, which reasons the applied design choices, is presented in Section 3.3. Finally, a comparison to other state-of-the-art NR-IQA methods is carried out in Section 3.4.

### 3.1. Evaluation Protocol

A reliable way to evaluate objective NR-IQA methods is based on measuring the correlation strength between the ground-truth scores of a publicly available IQA database and the predicted scores. In the literature, Pearson’s linear correlation coefficient (PLCC) and Spearman’s rank-order correlation coefficient (SROCC) are widely applied to characterize the degree of correlation. PLCC between vectors **x** and **y** can be expressed as

(18) $$PLCC\left(x,y\right)=\frac{\sum_{i=1}^{m}\left(x_{i}-\bar{x}\right)\left(y_{i}-\bar{y}\right)}{\sqrt{\sum_{i=1}^{m}{\left(x_{i}-\bar{x}\right)}^{2}}\sqrt{\sum_{i=1}^{m}{\left(y_{i}-\bar{y}\right)}^{2}}}$$

where $\bar{x}=\frac{1}{m}\sum_{i=1}^{m}x_{i}$ and $\bar{y}=\frac{1}{m}\sum_{i=1}^{m}y_{i}$. Furthermore, **x** stands for the vector containing the ground-truth scores, while **y** vector consists of the predicted scores. SROCC between vectors **x** and **y** can be defined as

(19) SROCC(x,y)=PLCC(rank(x),rank(y))

where the rank(·) function gives back a vector whose *i*th element is the rank of the *i*th element in the input vector. As a consequence, SROCC between vectors **x** and **y** can also be expressed as

(20) $$SROCC\left(x,y\right)=\frac{\sum_{i=1}^{m}\left(x_{i}-\hat{x}\right)\left(y_{i}-\hat{y}\right)}{\sqrt{\sum_{i=1}^{m}{\left(x_{i}-\hat{x}\right)}^{2}}\sqrt{\sum_{i=1}^{m}{\left(y_{i}-\hat{y}\right)}^{2}}}$$

where $\hat{x}$ and $\hat{y}$ stand for the middle ranks of **x** and **y**, respectively. Furthermore, the proposed algorithm and other learning-based state-of-the-art methods were evaluated by 5-fold cross-validation with 20 repetitions. Moreover, average PLCC and SROCC values are reported in this study.

### 3.2. Experimental Setup

ESPL-LIVE HDR [48], KADID-10k [49], CSIQ [50], TID2013 [39], and TID2008 [51] publicly available IQA databases were used to train and test the proposed algorithm. Table 2 illustrates some facts about the publicly available IQA databases used in this paper. It allows comparisons between the number of reference and test images, image resolutions, the number of distortion levels, and the number of distortion types. KADID-10k [49], CSIQ [50], TID2008 [51], and TID2013 [39] consist of a small set of reference images and artificially distorted images derived from the reference images using different distortion intensity levels and types. In contrast, ESPL-LIVE HDR [48] contains high dynamic range images created by multi-exposure fusion, tone mapping, or post-processing.

Specifically, the applied IQA database was divided randomly into a training set (∼80% of images) and a test set (∼20% of images) according to the reference images so no semantic content overlap was between these two sets. Moreover, average PLCC and SROCC values are reported measured over 20 random train-test splits.

### 3.3. Parameter Study

**Figure 5:**
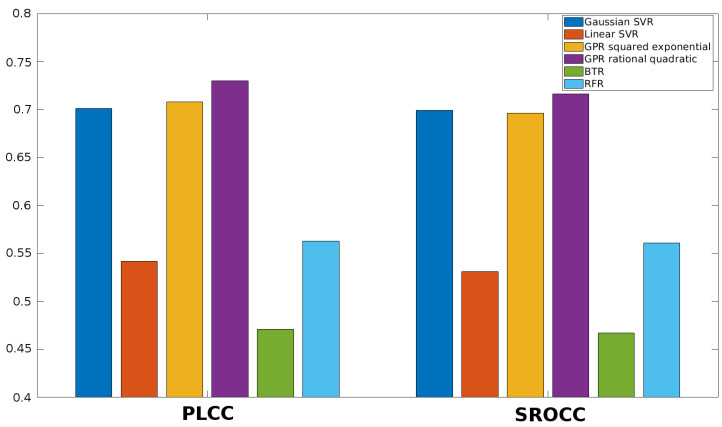
Comparison of different regression methods (SVR with Gaussian kernel function, SVR with linear kernel function, GPR with squared exponential kernel function, GPR with rational quadratic kernel function, BTR, and RFR). Mean PLCC and SROCC values were measured over 20 random train-test splits on KADID-10k [49].

**Figure 6:**
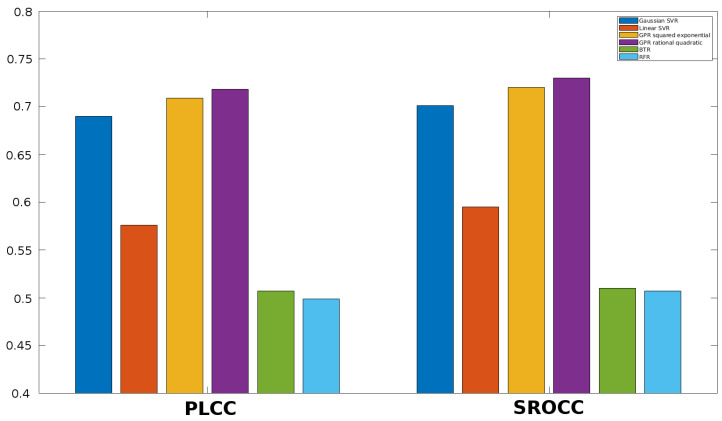
Comparison of different regression methods (SVR with Gaussian kernel function, SVR with linear kernel function, GPR with squared exponential kernel function, GPR with rational quadratic kernel function, BTR, and RFR). Mean PLCC and SROCC values were measured over 20 random train-test splits on ESPL-LIVE HDR [48].

**Figure 7:**
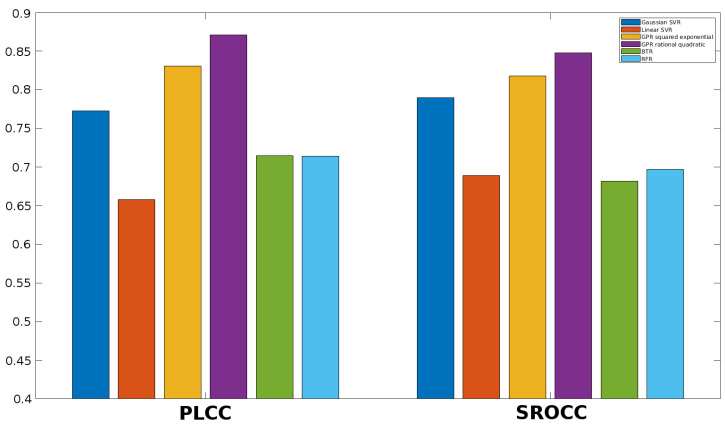
Comparison of different regression methods (SVR with Gaussian kernel function, SVR with linear kernel function, GPR with squared exponential kernel function, GPR with rational quadratic kernel function, BTR, and RFR). Mean PLCC and SROCC values were measured over 20 random train-test splits on CSIQ [50].

**Figure 8:**
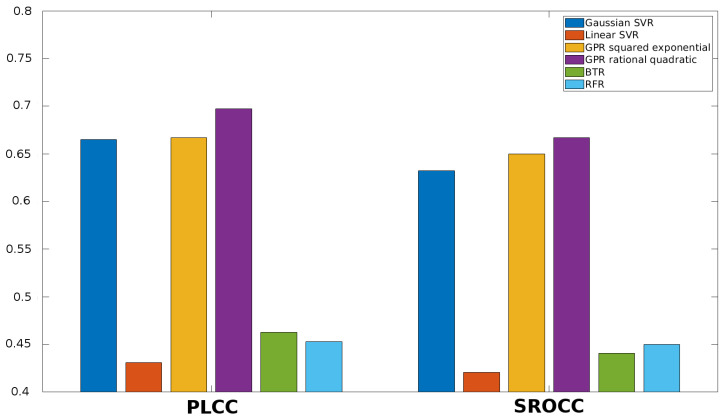
Comparison of different regression methods (SVR with Gaussian kernel function, SVR with linear kernel function, GPR with squared exponential kernel function, GPR with rational quadratic kernel function, BTR, and RFR). Mean PLCC and SROCC values were measured over 20 random train-test splits on TID2013 [39].

**Figure 9:**
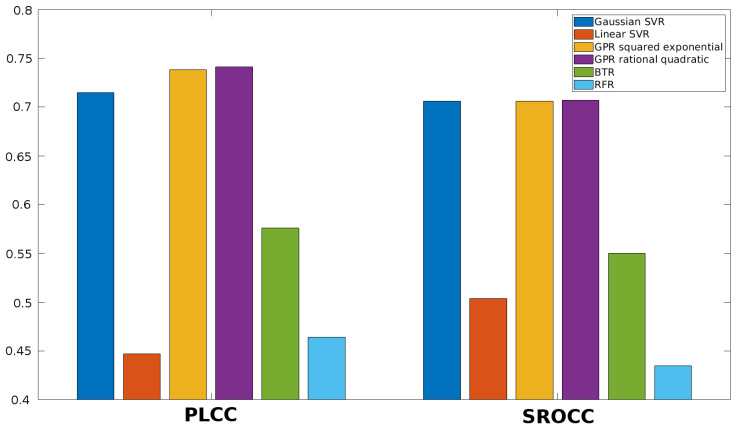
Comparison of different regression methods (SVR with Gaussian kernel function, SVR with linear kernel function, GPR with squared exponential kernel function, GPR with rational quadratic kernel function, BTR, and RFR). Mean PLCC and SROCC values were measured over 20 random train-test splits on TID2008 [51].

Once the feature vector is obtained, it must be mapped onto perceptual quality scores. Different machine learning techniques can be used to this end. First, the performance of different regression methods is examined in this section. More specifically, SVR with Gaussian kernel function, SVR with linear kernel function, Gaussian process regression (GPR) with squared exponential kernel function, GPR with rational quadratic kernel function, binary tree regression (BTR), and random forest regression (RFR) are considered in this study. The results for KADID-10k [49], ESPL-LIVE HDR [48], CSIQ [50], TID2013 [39], and TID2008 [51] databases are summarized in Figure 5, Figure 6, Figure 7, Figure 8 and Figure 9. From these results, it can be clearly seen that GPR with rational quadratic kernel function significantly outperforms the other examined regression techniques. As a consequence, GPR with rational quadratic kernel function was used in the further experiments.

Table 3 and Table 4 illustrate the performance of the proposed method over different distortion intensity levels and types of TID2013 [39] and TID2008 [51], respectively. From these results, it can be seen that the proposed method performs better on higher distortion levels. Furthermore, it can be seen that JPEG transmission errors, non-eccentricity pattern noise, and mean shift are very challenging distortion types, while on JPEG and JPEG2000 compressed images the proposed method achieves very high performance.

### 3.4. Comparison to the State-of-the-Art

The proposed algorithm—codenamed SPF-IQA—was compared to several state-of-the-art NR-IQA algorithms (BIQI [15], BLIINDS-II [17], BRISQUE [20], CORNIA [52], CurveletQA [26], DIIVINE [53], HOSA [54], FRIQUEE [25], GRAD-LOG-CP [23], IQVG [55], PIQE [56], SSEQ [57], and NBIQA [28]) whose original source codes are available online. These methods were re-trained using exactly the same database partition that was applied for the proposed method. As already mentioned, the used IQA database was randomly split into a training set (∼80% of images) and a test set (∼20% of images) according to the reference images. As a consequence, no semantic content overlap was between these two sets. Moreover, mean PLCC and SROCC values are reported measured over 20 random train-test splits. Besides the average PLCC and SROCC values, the statistical significance is also reported following the guidelines of [58]. As recommended in [58], the variance of *z*-transforms were estimated as 1.06/(N−3) where *N* stands for the number of images in a given database. Specifically, those correlation coefficients which are significantly different from SPF-IQA’s are highlighted with color in Table 5. The results of the performance comparison to the state-of-the-art on ESPL-LIVE HDR [48], KADID-10k [49], CSIQ [50], TID2013 [39], and TID2008 [51] are summarized in Table 5. From these results, it can be seen that the proposed method is able to significantly outperform the state-of-the-art on three large publicly available databases (ESPL-LIVE HDR [48], KADID-10k [49], and CSIQ [50]) in terms of PLCC and SROCC. On TID2008 [51], the proposed method is able to slightly outperform the best state-of-the-art algorithms. On TID2013 [51], SPF-IQA achieves the state-of-the-art but does not outperform the best method (FRIQUEE [25]). From Table 5, one can have another observation. SPF-IQA is superior to other state-of-the-art methods in terms of weighted PLCC and SROCC.

In Table 6, the computational times and feature vector lengths of the learning based algorithms were compared. It can be observed that the feature extraction procedure of SPF-IQA takes less than five other state-of-the-art methods (BLIINDS-II [17], DIIVINE [53], FRIQUEE [25], IQVG [55], NBIQA [28]). The computational times were measured on a personal computer containing 8-core i7-7700K CPU in MATLAB R2019a environment. On the other hand, the length of SPF-IQA’s feature vector is not significantly larger than those of other state-of-the-art methods.

## 4. Conclusions

In this paper, the application of statistical and perceptual features was studied for no-reference image quality assessment and a novel feature extraction method was also proposed. Statistical features incorporated local fractal dimension distribution, first digit distribution in the wavelet and spatial domain, and color statistics. On the other hand, powerful perceptual features, such as colorfulness, global contrast factor, dark channel feature, entropy, and mean of phase congruency image, were also utilized. On the whole, the proposed algorithm required only 52 statistical and 5 perceptual features. In a parameter study, a wide range of regression techniques was investigated to select the one which best fits to the proposed feature vector. Finally, a Gaussian process regression (GPR) model with rational quadratic kernel function was applied to create a mapping between the feature vectors and perceptual quality scores. Experimental results on five large publicly available databases (ESPL-LIVE-HDR, KADID-10k, CSIQ, TID2013, and TID2008) showed that the proposed method is able to outperform other state-of-the-art methods.

## Figures and Tables

**Table 1:** Mean and median distances from the Benford’s law in TID2013 [39] measured in the wavelet (horizontal, vertical, and diagonal detail coefficients) and spatial (gradient magnitude) domains.

	Mean	Median	Standard Deviation
Horizontal detail coefficients—Reference images	0.026	0.020	±0.019
Horizontal detail coefficients—Distorted images	0.043	0.029	±0.055
Vertical detail coefficients—Reference images	0.024	0.018	±0.020
Vertical detail coefficients—Distorted images	0.042	0.027	±0.056
Diagonal detail coefficients—Reference images	0.029	0.026	±0.018
Diagonal detail coefficients—Distorted images	0.049	0.036	±0.054
Gradient magnitudes—Reference images	0.069	0.053	±0.052
Gradient magnitudes—Distorted images	0.073	0.058	±0.050

**Table 2:** Comparison of publicly available IQA databases used in this study.

Database	Ref. Images	Test Images	Resolution	Distortion Levels	Number of Distortions
TID2008 [51]	25	1700	512×384	4	17
TID2013 [39]	25	3000	512×384	5	24
CSIQ [50]	30	866	512×512	4–5	6
ESPL-LIVE HDR [48]	-	1811	960×540	-	-
KADID-10k [49]	81	10,125	512×384	5	25

**Table 3:** Average PLCC and SROCC values of the proposed method for each distortion level of TID2013 [39] and TID2008 [51].

Distortion Level	TID2013 [39]		TID2008 [51]	
	PLCC	SROCC	PLCC	SROCC
Level 1	0.383	0.448	0.478	0.484
Level 2	0.355	0.423	0.452	0.494
Level 3	0.288	0.307	0.719	0.675
Level 4	0.592	0.560	0.791	0.758
Level 5	0.746	0.739	-	-
All	0.697	0.667	0.741	0.707

**Table 4:** Average PLCC and SROCC values of the proposed method for each distortion type of TID2013 [39] and TID2008 [51].

Distortion Type	TID2013 [39]		TID2008 [51]	
	PLCC	SROCC	PLCC	SROCC
Additive Gaussian noise	0.763	0.798	0.849	0.836
Additive noise in color components	0.572	0.813	0.599	0.699
Spatially correlated noise	0.963	0.969	0.899	0.914
Masked noise	0.425	0.532	0.385	0.495
High frequency noise	0.929	0.930	0.925	0.922
Impulse noise	0.586	0.619	0.745	0.761
Quantization noise	0.673	0.707	0.593	0.687
Gaussian blur	0.699	0.707	0.681	0.693
Image denoising	0.594	0.520	0.672	0.654
JPEG compression	0.877	0.972	0.835	0.866
JPEG2000 compression	0.913	0.872	0.848	0.817
JPEG transmission errors	0.003	0.080	0.031	0.108
JPEG2000 transmission errors	0.481	0.513	0.342	0.571
Non eccentricity pattern noise	0.007	0.044	0.001	0.009
Local block-wise distortions	0.600	0.673	0.784	0.847
Mean shift	0.038	0.001	0.258	0.012
Contrast change	0.409	0.416	0.361	0.444
Change of color saturation	0.771	0.757	-	-
Multiplicative Gaussian noise	0.931	0.990	-	-
Comfort noise	0.240	0.302	-	-
Lossy compression of noisy images	0.915	0.931	-	-
Image color quantization with dither	0.822	0.879	-	-
Chromatic aberrations	0.362	0.352	-	-
Sparse sampling and reconstruction	0.890	0.792	-	-
All	0.697	0.667	0.741	0.707

**Table 5:** Comparison to the state-of-the-art on ESPL-LIVE HDR [48], KADID-10k [49], CSIQ [50], TID2013 [39], and TID2008 [51]. Mean PLCC and SROCC values were measured over 20 random train-test splits. The best results are typed in **bold**. The green background color stands for that the correlation is lower than those of the proposed method and the difference is statistically significant with *p* < 0.05, while the red background color means the correlation is higher and the difference is statistically significant with *p* < 0.05.

	ESPL-LIVE HDR [48]		KADID-10k [49]		CSIQ [50]		TID2013 [39]		TID2008 [51]		Weighted Average	
Method	PLCC	SROCC	PLCC	SROCC	PLCC	SROCC	PLCC	SROCC	PLCC	SROCC	PLCC	SROCC
BIQI [15]	0.476	0.405	0.463	0.418	0.560	0.524	0.521	0.392	0.537	0.396	0.486	0.415
BLIINDS-II [17]	0.459	0.448	0.567	0.521	0.816	0.774	0.554	0.560	0.562	0.570	0.565	0.537
BRISQUE [20]	0.446	0.423	0.531	0.528	0.763	0.704	0.477	0.418	0.492	0.423	0.521	0.497
CORNIA [52]	0.684	0.624	0.588	0.551	0.809	0.711	0.710	0.612	0.699	0.608	0.641	0.582
CurveletQA [26]	0.547	0.544	0.501	0.467	0.631	0.584	0.554	0.503	0.598	0.562	0.531	0.496
DIIVINE [53]	0.483	0.489	0.529	0.470	0.793	0.768	0.689	0.599	0.698	0.610	0.581	0.522
HOSA [54]	0.691	0.683	0.625	0.620	0.821	0.797	0.760	0.675	0.725	0.701	0.674	0.653
FRIQUEE [25]	0.657	0.623	0.657	0.643	0.818	0.770	**0.773**	**0.706**	0.728	0.699	0.692	0.663
GRAD-LOG-CP [23]	0.540	0.540	0.501	0.467	0.834	0.821	0.669	0.635	0.713	**0.716**	0.571	0.545
IQVG [55]	0.408	0.416	0.552	0.534	0.796	0.753	0.596	0.524	0.631	0.591	0.564	0.536
PIQE [56]	0.025	0.033	0.289	0.237	0.644	0.522	0.462	0.364	0.427	0.327	0.322	0.261
SSEQ [57]	0.482	0.505	0.381	0.401	0.783	0.707	0.659	0.574	0.664	0.563	0.486	0.472
NBIQA [28]	0.655	0.658	0.632	0.625	0.834	0.799	0.720	0.674	0.724	0.697	0.668	0.652
SPF-IQA (proposed)	**0.718**	**0.730**	**0.715**	**0.707**	**0.871**	**0.848**	0.697	0.667	**0.741**	0.707	**0.722**	**0.710**

**Table 6:** Average feature extraction time comparison of learning-based NR-IQA methods measured on KADID-10k [49].

Method	Length of the Feature Vector	Time Cost (seconds)
BIQI [15]	18	0.011
BLIINDS-II [17]	24	20.200
BRISQUE [20]	36	0.020
CORNIA [52]	100	0.075
CurveletQA [26]	12	0.542
DIIVINE [53]	88	5.845
FRIQUEE [25]	560	7.807
GRAD-LOG-CP [23]	40	0.014
HOSA [54]	14,700	0.141
IQVG [55]	8000	12.970
SSEQ [57]	12	0.486
NBIQA [28]	51	7.775
SPF-IQA (proposed)	57	2.783
